# High-resolution mapping of injury-site dependent functional recovery in a single axon in zebrafish

**DOI:** 10.1038/s42003-020-1034-x

**Published:** 2020-06-12

**Authors:** Alexander Hecker, Pamela Anger, Philipp N. Braaker, Wolfram Schulze, Stefan Schuster

**Affiliations:** Department of Animal Physiology, University of Bayreuth, 95440 Bayreuth, Germany

**Keywords:** Spinal cord injury, Regeneration and repair in the nervous system

## Abstract

In non-mammalian vertebrates, some neurons can regenerate after spinal cord injury. One of these, the giant Mauthner (M-) neuron shows a uniquely direct link to a robust survival-critical escape behavior but appears to regenerate poorly. Here we use two-photon microscopy in parallel with behavioral assays in zebrafish to show that the M-axon can regenerate very rapidly and that the recovery of functionality lags by just days. However, we also find that the site of the injury is critical: While regeneration is poor both close and far from the soma, rapid regeneration and recovery of function occurs for injuries between 10% and 50% of total axon length. Our findings show that rapid regeneration and the recovery of function can be studied at remarkable temporal resolution after targeted injury of one single M-axon and that the decision between poor and rapid regeneration can be studied in this one axon.

## Abstract (web summary)

Alexander Hecker et al. study the regeneration potential of the axon of the giant Mauthner (M) neuron in zebrafish. Using two-photon microscopy and behavioral assays, they show that the M-axon can recover rapidly days after injury. They also characterize the optimal injury site that enables rapid regeneration and functional recovery.

## Introduction

The central nervous system of humans and other mammals is unable to regenerate injured axons on its own. One of the main reasons for this is the inability of the axon to penetrate the hostile and nonpermissive extracellular barrier that forms after spinal cord injury (SCI)^1–3^. Several ways are pursued that facilitate regeneration across the dystrophic barrier by using grafts^2,4^ or by various forms of modulating the hostile interaction e.g., through chondroitinase-ABC^5,6^, targeting inflammatory mediators involved in CNS proliferation^7^, inhibiting transcription factors^8^, or stem cell transplantation^9^. Additional useful approaches might emerge from the study of regeneration in the CNS of nonmammalian vertebrates, such as fish or amphibians^10–14^. Some, but not all, neurons of their CNS demonstrate significant spontaneous recovery after SCI^14,15^ and in general have a higher capacity for regeneration than mammals^16,17^. Moreover, fish and amphibians offer ways for studying the vast range of processes that must come packaged with axonal regrowth^18^ to restore function. For instance, the disconnected distal part of the axon first needs to undergo Wallerian degeneration^19,20^ and resulting cell debris needs to be removed^21,22^, while at the same time decay of the proximal axon and soma needs to be prevented. The remaining axon must then initiate targeted and robust axon growth^23^, but then also needs to be re-myelinated and properly connected to its target motoneurons, which requires targeted synaptogenesis and adjustment in the synaptic weights.

Several studies have successfully monitored the recovery of behavioral function after spinal cord injury in fish and amphibians. Among the many axons affected by SCI the large axons of the two Mauthner (M-) cells are easiest to recognize from their unique diameter and so are easiest to track. Doing so, studies in early zebrafish larvae^24^, in adult zebrafish^14^, in goldfish^25^, lamprey^26^ in adult salamanders^27^ indicate slow regeneration of the M-axon. In lamprey behavioral recovery occurred after 6 weeks^28^ but often only after months^29^. In adult goldfish recovery of motor behaviors usually took up to 2 months^30^ but, surprisingly, escape latency never fully recovered even within one year. In adult zebrafish, swimming distance recovered within 6–8 weeks^31^. One study used confocal microscopy in zebrafish larvae to track the regeneration of spinal axons after distal SCI in zebrafish larvae and showed that the M-axon belongs to the group of axons that regenerated poorly and that its regrowth required stimulation by additional doses of cAMP^24^.

We found these findings puzzling from a neuroethological point of view. Why should an axon that is involved in life-saving escape behavior show poorer regeneration than other axons available to the animal? This problem gets even more pressing in light of recent findings that show that the M-axon is required for short-latency escapes, that not having the M-axon reduces chances to survive the attacks of a natural predator and that absence of the M-axon can never be completely compensated^32^. So, it would be implausible that specifically the M-axon should poorly regenerate after systemic injury.

We therefore decided to re-examine the regenerative capacities of the M-axon. Our approach has been triggered by a range of studies^33–40^, that clearly revealed an uneven distribution of regenerative capacities across a nerve. Specifically, these studies showed that regenerative capacity diminishes the farther away from the somata the nerve had been crushed. Perhaps, similarly, also the M-axon might be able to regenerate quickly, but only after injuries closer to its soma. Zebrafish are ideally suited to test this hypothesis using appropriate zebrafish lines and two-photon microscopy to specifically damage only the M-axon at a precisely targeted distance from its soma and then to follow subsequent regeneration of the axon over days^32^. Moreover, should the axon show sufficiently rapid regeneration, then the recent finding of a unique association of the M-axon and the short-latency escapes of larval and adult zebrafish^32^ offers an exciting possibility: Targeted injury of the axon of one specific neuron—unusually—should lead to a escape phenotype whose recovery could then uniquely be attributed to the recovery of a single neuron, its post-injury remyelination and synaptogenesis.

In this paper we demonstrate that such an approach is possible: Exploiting two-photon microscopy we show that the M-axon does not regenerate poorly per se, as previously reported^14,24–27,33^, but that it rather can regrow very rapidly with crucial aspects of its function also completely restored in days. The discrepancy to earlier findings is explained from the fact that, similarly as in the rodent optic nerve, regenerative capacity of the M-axon is not distributed homogeneously and is worse both after injury very close to the soma and very far from it. Hence the M-cell of zebrafish is a powerful model to study the nature of positional effects in axon regeneration and—because of its unique association with short-latency escapes—to monitor functional recovery after targeted injury of one single axon at a temporal resolution of less than one day.

## Results

### Proximity of injury site to the soma determines regeneration

**Fig. 1:**
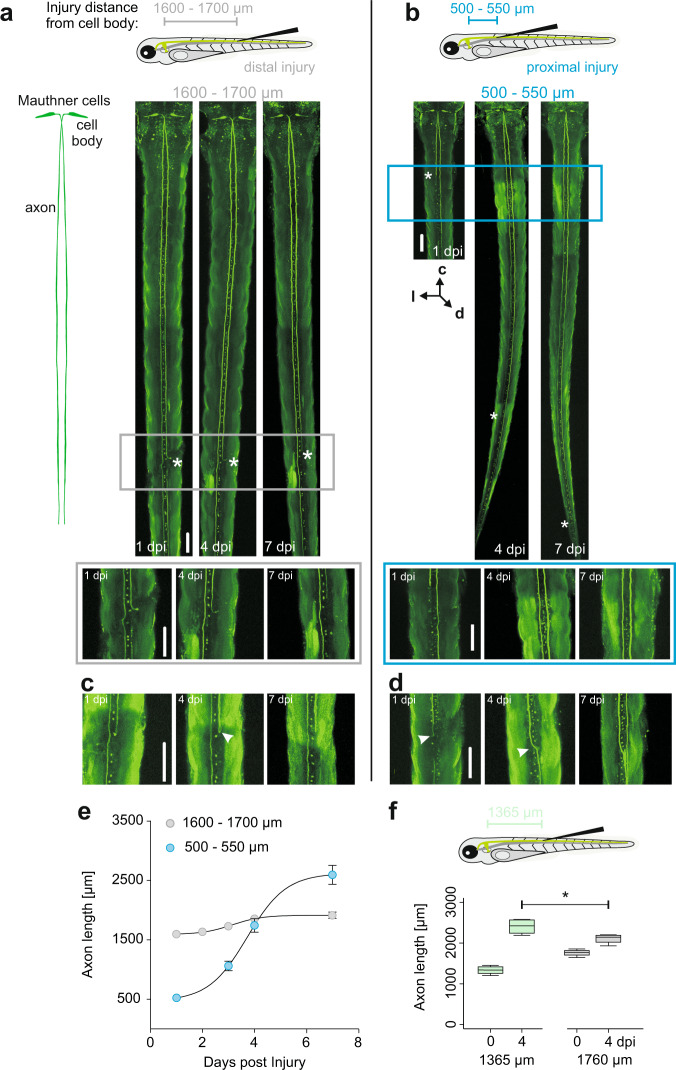
Proximity of systemic injury to the soma decides whether the giant Mauthner axon regenerates or not. **a**, **b** show projections of two-photon (2P−) laser z-stacks of an injured M-axon one, four, and seven days after either distal (**a**) or proximal (**b**) injury. Outlines of the M-cell and its axon are shown on the left. Systemic injuries were set using a broken glass microelectrode (top panels). Rectangular boxes and zoomed in images below highlight differences in axonal regrowth after distal and proximal injury. Asterisks indicate most caudal end of the axon. Orientation is shown in **b** (c = cranial; l = lateral; d = dorsal). Scale bar = 100 μm. **a** Example of a distally injured M-axon. Left axon remained intact, the right axon was injured but did not grow past the injury site. **b** Example of a proximally injured M-axon. The injured axon showed robust regeneration through the injury site. **c**, **d** show examples of aberrant regeneration after distal (**c**) and proximal (**d**) systemic spinal cord injury. Scale bar = 100 μm. **c** After distal injury the axon changed direction (U-bend, see arrow) and regenerated cranially. **d** After proximal injury aberantly regrown axons always pass the site of injury and grow in the appropriate direction, but occasionally switch side (arrow). Note that the aberrant axon still shows robust regeneration. **e** Quantification of axon regeneration over time. Axon length was monitored one, two, three, four and seven dpi after distal (grey) or proximal (blue) injury. Data are shown as mean ± SEM. Nonlinear sigmoidal fits (logistic function) are shown. Distal: R^2^ = 0.24; *N* = 28; relative steepness s = 0.7; maximal slope at x_0_ = 3.1 days. Proximal: R^2^ = 0.73; *N* = 24; s = 0.58; x_0_ = 3.7 days. **f** Axon length of siblings after systemic injury 1365 ± 38 μm and 1760 ± 38 μm from the soma either directly post-injury (0 dpi) or 4 dpi (*N* = 4). Significant difference is highlighted with an asterisk (*p* = 0.04, Mann–Whitney-U test) between axon lengths at 4 dpi.

**Fig. 2:**
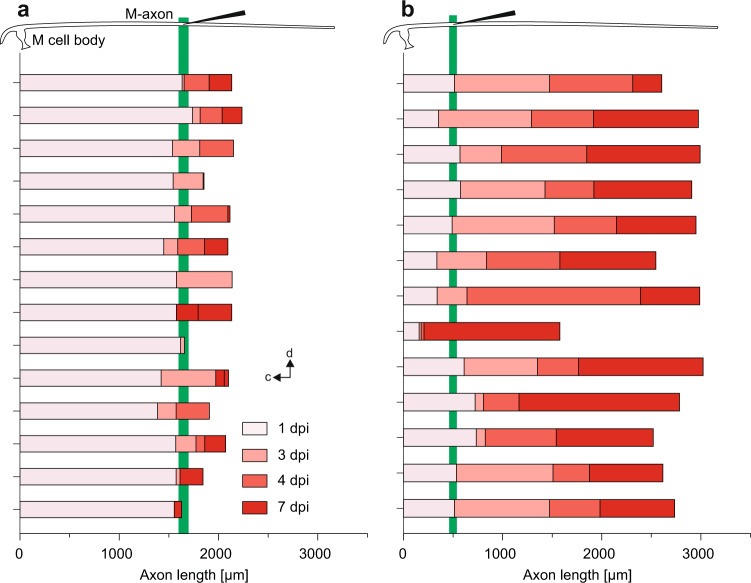
The degree of variation in axon regeneration after systemic spinal cord injury. **a**, **b**: Diagrams show the regrowth of individual Mauthner cell axons in individual larvae after either distal (**a**) or proximal (**b**) systemic spinal cord injury. Injury site is indicated in M-cell schematic (top) and by green vertical bar. The color code marks the axon length reached at the indicated time and is additionally assigned to the interval that follows after the last measurement (i.e., dark red ‘7 dpi’ indicates length measured at 7 dpi and shows increment in length after the last measurement at 4 dpi). 0 μm is where the axon emerges from the soma. Orientation of the M-cell is as indicated (d: dorsal; c: cranial). Note the initial retraction of most axons at 1 dpi seen in **a** and in some, but not all, axons in **b**.

We first examined the possibility that the Mauthner (M-) axon might regenerate better when the spinal cord is injured closer to the soma of the M-cell. We therefore compared regeneration of the M-axon in zebrafish larvae after systemic spinal cord injury had occurred either distally, as in a previous work^24^, or much closer to the soma (proximal injury; 500–550 μm from the soma; Fig. 1a, b). Following distal injury (1599 ± 31 μm; *N* = 28), the severed caudal part of the M-axon always (14 of 14 cases) degenerated within 24 h through Wallerian degeneration^19,41^. The rostral part of the M-axon that was still in contact with its soma often withdrew a few micrometers from the injury site but always survived. However, only 46% (13 out of 28) of the M-axons showed a regenerative response, thus confirming previous findings^24^. Moreover, regeneration, when it occurred, was poor (Fig. 1a, e; Fig. 2a; Axon lengths: 2 dpi: 1637 ± 52 μm; 3 dpi: 1731 ± 40 μm; 4 dpi: 1858 ± 47 μm; 7 dpi: 1915 ± 60 μm). In many cases (13 of 28) the axon was unable to penetrate the injury site and axons showed a phase in which they grew aberrantly and even in reversed (anterior) direction (Fig. 1c). Such aberrations indicate that axonal regrowth per se might still be possible but that targeted regeneration is inhibited, making the recovery of function impossible.

While the results obtained after distal systemic injury thus fully support earlier findings^24^, our results after proximal injury show that the M-cell does not generally lack the ability to regenerate and that the distance of the injury site from the soma determines whether robust regeneration occurs or not. In all M-cells (24 of 24) in which we set systemic spinal cord injury in proximal positions, the axons were fully capable of robustly regrowing through the site of injury (Fig. 2b). Almost all axons showed a distinct regenerative response within three days post-injury (dpi), after which time average axon length had doubled from its initial length post-injury (523 ± 31 μm; *N* = 24 larvae) to a length of 1061 ± 78 μm (Fig. 1b, e). One day later (4 dpi) the axons had regrown to an average length of 1742 ± 111 μm and at 7 dpi had achieved the normal length (2593 ± 148 μm) that axons of their untreated siblings had at the same age. After proximal injury we also noticed cases with aberrant axonal regrowth; however, these were distinctly different from the aberrant regrowth seen after distant injury, in which axons grew in the opposite direction (after forming u-bends). After proximal injury regrowth in the wrong direction never occurred, but in some cases (7 of 24) the regrowing axon crossed the midline to then regenerate on the same side as the axon of the other M-cell (see Fig. 1d). We additionally injured the spinal cord at an intermediate distance, 1365 ± 38 μm from the soma (Fig. 1f). Here, regeneration was also robust, i.e., 4 dpi axons were significantly longer than axons from siblings with distant injuries (1760 ± 38 μm; N = 4; Mann–Whitney-U test). Summed up, we discovered that M-axons are not generally restricted in their capacity to regenerate after systemic spinal cord injury. They even regenerate at a remarkable speed of a few days, depending on where the injury occurred.

### M-axon specific injury confirms extremely rapid regeneration

**Fig. 3:**
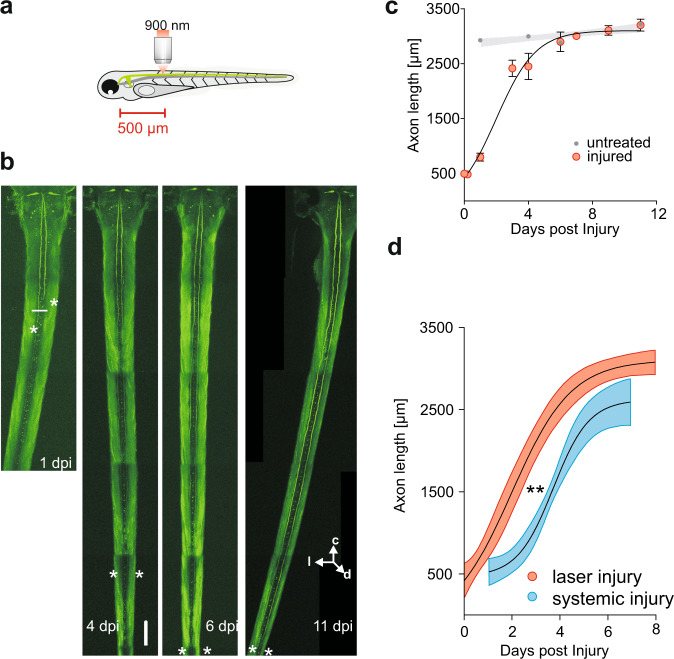
Targeted proximal injury reveals rapid axonal regeneration in the Mauthner cell. **a** Schematic illustration of Mauthner (M)-cell specific laser (900 nm) axotomy in *Ca-Tol-056* larvae (with GFP-filled M-cells) approximately 500 μm away from the M-soma. **b** Projection of two-photon-laser z-stack of injured M-axons 1, 4, 6, and 11 days post-injury (dpi). Asterisk depicts most caudal axon stump. White line shows injury site. Orientation is indicated in the bottom right (c = cranial; l = lateral; d = dorsal). Scale bar: 100 μm. **c** Quantification of axon regeneration over time. Data are shown as Mean (±SEM). Regrowth is well described by a sigmoidal function (R^2^ = 0.76; *N* = 16) with slope parameter (steepness) s = 0.4 and maximal slope at x_0_ = 2 days. Gray area indicates axon growth in untreated siblings over the same period (*N* = 8 M-cells). **d** Comparison of time course of regeneration after systemic and after targeted M-axon injury. Plot compares sigmoidal fit of M-axon regeneration after proximal systemic spinal cord injury (Fig. 1e) and proximal laser-induced M-axon injury (from c) with respective 95% confidence bands. Steepness of the two sigmoidal curves is not significantly different (*P* = 0.2), but time of maximal regrowth is significantly later after systemic injury (*P* = 0.007; extra sum-of-square F test).

**Fig. 4:**
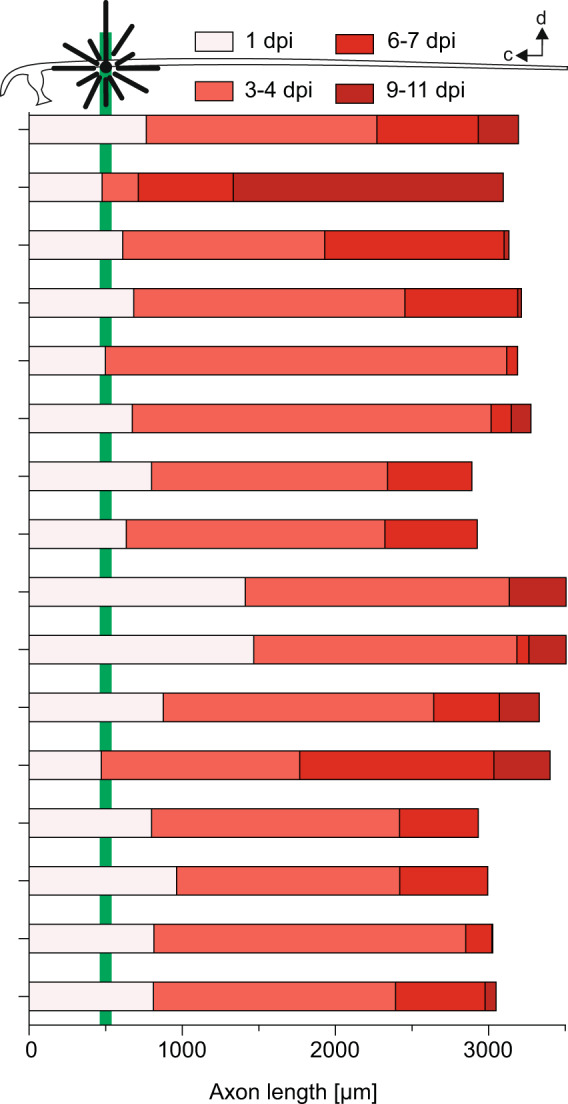
The variations in the time course of axonal regrowth following targeted proximal M-axon injury. The length over time of individual M-axons after laser-induced proximal injury of the GFP-filled axons (injury site indicated in M-cell schematic and green bar). Color code indicates length reached at indicated time as well as increment from previous measurement (e.g., darkest red color indicates length measured between 9 and 11 dpi and highlights growth after 6 and 7 dpi). 0 μm is where the axon emerges from the soma. Orientation of the M-cell schematic is shown in the top right (d: dorsal; c: cranial).

In the systemic injuries set in the previous experiments, the spinal cord was physically transected with a glass micropipette (see Methods). This means that skin, muscles and many other neurons of the CNS were injured in addition to the M-axon. In other words, systemic injury also influences many factors outside the neuron whose specific regeneration is then followed. Targeted M-axon injury using a two-photon microscope, in contrast, would affect specifically a single axon. Because of the unique relation between the functionality of a single M-axon and the occurrence of short-latency escapes^32^ it should even be possible to follow the regeneration of the M-axon while in parallel observing short-latency escapes to probe the time course of functional recovery—thus linking functional recovery with the regeneration of a single axon. Precisely targeted and M-axon specific injury was achieved by using the GFP labelled M-cells of our *Ca-Tol-056* line and a two-photon laser^42^. First, we confirmed the regenerative capacity of the M-axon for injury in approximately 500 μm (495 ± 17 μm; *N* = 16) distance from the M-soma (Fig. 3a, also see SM1). The basic characteristics of regrowth after M-axon specific laser-induced injury were remarkably similar to those observed after systemic SCI, including Wallerian degeneration of the severed caudal part of the axon and an initial short-range retraction of the surviving soma-connected axon stump. Already 1 day post-injury M-axons were grown to a length of 797 ± 70 μm. Remarkably, between three and four dpi, axons regenerated to a length of 2435 ± 150 μm (*N* = 16) and had completed regeneration within 6 days (2897 ± 169; *N* = 16 M-axons; Fig. 3b, c). After this time axon length was just as that in untreated larvae at the same age (see gray area in Fig. 3c). In marked contrast to systemic SCI, the regrowing M-axons always robustly penetrated the injury site after laser-induced injury (Fig. 4), and then grew sigmoidally (logistic function; *N* = 16; R^2^ = 0.76) between one and six dpi (Fig. 3c). Furthermore, none of the aberrations (e.g., side crossing) seen after systemic SCI were seen after targeted M-axon specific injury.

We next compared the phase of sigmoidal growth of the M-axon after systemic SCI and after laser-induced M-axon specific injury. Figure 3d shows the two nonlinear fits after proximal systemic SCI (from Fig. 1e) and after laser-induced proximal injury (Fig. 3c). We additionally plotted the 95% confidence band of the sigmoidal curves and statistically compared their best-fit values. Maximal steepness of the curves showed no significant difference (*P* = 0.2; Extra sum-of-square F test), suggesting that once regeneration is initiated it proceeds similarly, regardless of how the injury occurred. However, the timing of maximal regrowth (i.e., the point of maximal slope of the sigmoid curve) was significantly delayed by about 1.7 days after systemic spinal cord injury (systemic SCI: 3.7 ± 0.2 days; laser-induced injury: 2 ± 0.5 days; *p* = 0.007, Extra sum-of-square F test). In other words, these experiments confirm the remarkable regenerative capability of the M-axon after proximal injury and show that it is even faster than found after systemic injury, being fully completed after only 6 days.

### Mapping regenerative capacity across the M-axon

**Fig. 5:**
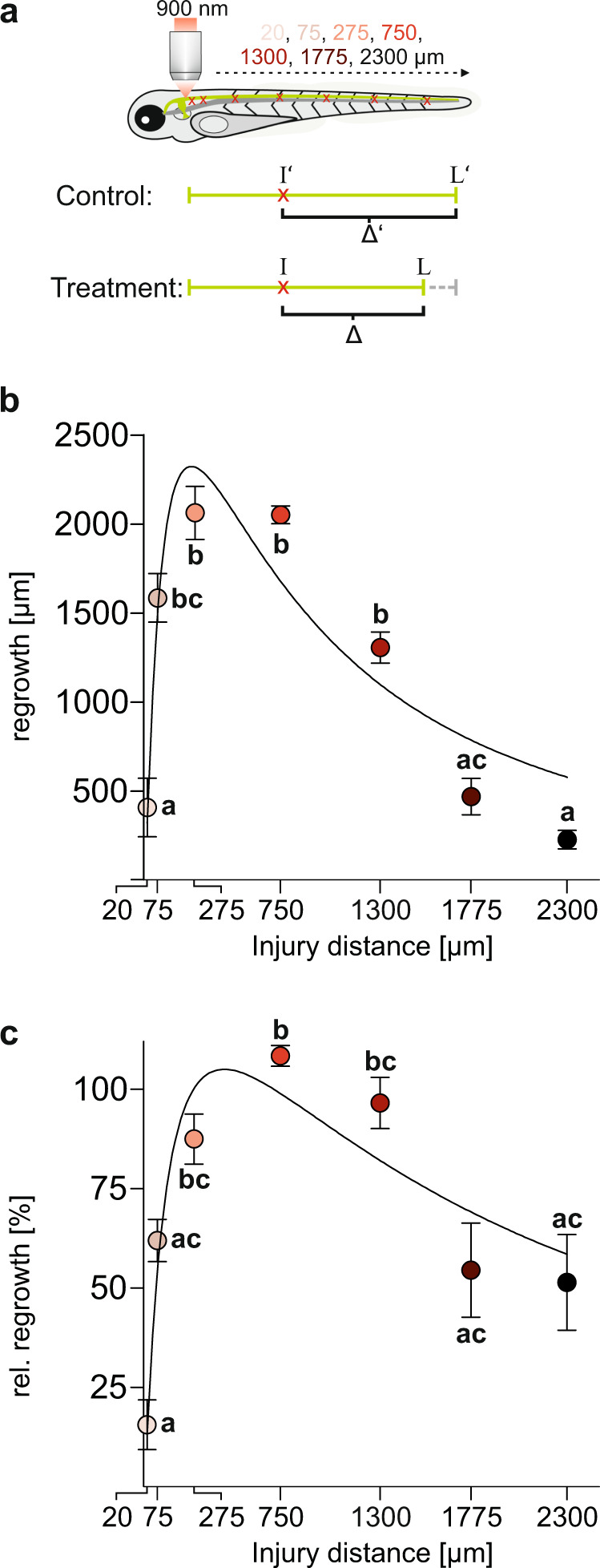
High-resolution mapping of regenerative capacity across the length of the Mauthner axon. **a** Targeted laser-induced injury allows to specifically injure the Mauthner axon at specific points along its length, starting very close to the soma. Regrowth can then be monitored over several days and compared with axon length in untreated siblings (control). **b** Absolute regrowth Δ on 4 dpi across all injury distances. Data are reasonably well described by a logarithmic Gaussian fit (R^2^ = 0.66), with an optimum regenerative efficiency at an injury distance of about 258 ± 19 μm, i.e., at about 10% of total axon length. **c** We also considered relative regrowth (100*Δ/Δ′), i.e., absolute regrowth normalized to average fictive regrowth Δ′ (for a virtual injury at the same distance) in siblings of equal age. Relative regrowth also shows the clear dependence of regeneration on the soma-distance and the decline at large distances (R^2^ = 0.49, with optimum regenerative efficiency at 438 ± 46 μm). **b**, **c** Significant differences, denoted by different letters, were estimated with the Kruskal–Wallis test followed by Dunn’s multiple comparison test (*p* < 0.0001). Circles depict mean ± SEM (*N* = 10–16).

To map the speed of M-axon regeneration after precisely targeted injury at various distances from the soma in more detail we used the advantages of the two-photon laser. This allowed us to injure the M-axon at a range of precisely set distances, including some very close to the soma that would have been impossible to explore with systemic injury (as it would severely damage the brain). First, we studied the consequences of injuries very close to the soma. When we injured the M-axons as close as 19 ± 0.3 μm (*N* = 24) to its soma (see Supplementary fig. 1), the somata died in 25% of all cases. Furthermore, in those cases in which the soma survived, regeneration did not occur in 9 out of 16 axons and the axon remained absent (after an initial retraction) for at least four dpi (e.g., see Bottom panel Supplementary fig. 1). When we injured the axon at 75 ± 1 μm (*N* = 16) a smaller fraction of the somata (6.25%) died and at an injury distance of 250 μm the soma never died (*N* = 16). Hence, at very close proximity to the soma axon injury can lead to death of the soma so that no subsequent M-axon recovery is possible. Next, we injured the M-axon at six other distances from the soma, between 75 and 2300 μm (Fig. 5a; 75 ± 1 μm, 275 ± 1 μm, 743 ± 2 μm, 1283 ± 2 μm, 1775 ± 4 μm & 2296 ± 7 μm; *N* = 16 axons for each distance). In total we thus cover 1–90 % of the length of the M-axon at the age when injuries were made. We then monitored the regrowth every day from 1 dpi to 4 dpi.

To compare the regenerative efficiency of each injury distance, we determined axon regrowth (Δ) at 4 dpi (Fig. 5b). The results are striking and clearly reveal the position-dependency with two major soma-distance-dependent factors: Both very close to the soma (i.e., <75 μm) and at large distance (i.e., >1300 μm) absolute regrowth is small. Approximating a log-Normal distribution (R^2^ = 0.66) yielded an estimate of 258 ± 19 μm for the distance at which regeneration was optimal, but clearly there is a broad range from about 75–1300 μm. While the situation is clear at close distances we wondered whether simply reporting the actual regrowth would underestimate the regenerative capacity of the axon at distal positions. This could happen, for instance, if unknown regulatory mechanisms would work like a proportional controller and adjust the speed of regeneration such that it is adjusted to the mismatch between the intended (normal) axon length and the actual one after injury. This would clearly introduce an apparently lower regrowth for the distal injuries. To account for this possibility, we also considered a measure, called relative regrowth, by normalizing the absolute regrowth to the regrowth (Δ′) that would maximally be possible. The latter was estimated by taking the average axon length of untreated siblings of the same age (*N* = 8) and subtracting the respective injury distance (Fig. 5a, c). We would like to emphasize that there is no evidence in the M-axon of regeneration speed being proportional to the mismatch (Δ′). But considering relative regrowth is important in order not to overestimate the positional dependent effect. However, even when considering relative regrowth, the decline at large distances can still be seen. Relative regrowth at 4 dpi also showed optimal regeneration at intermediate distances from the soma and optimal distance could be estimated by fitting a log-Gaussian curve (optimum regeneration at 438 ± 46 μm; R^2^ = 0.49). At very close distances also relative regrowth declined (16 ± 6% at 20 μm; 62 ± 5% at 75 μm). At more distant injuries relative regrowth was also reduced (54 ± 12% at 1775 and 51 ± 12% at 2300 μm; Kruskal–Wallis test followed by Dunn’s multiple comparison test; *p* < 0.0001), while injury at 750 μm from soma showed good regeneration (108 ± 3%).

We would like to emphasize that the positional dependence of both absolute and relative regrowth can already be seen before 4 dpi (Fig. S2). As we measured axonal regrowth in each larva every day (by quickly embedding the larva, monitoring the axon under the two-photon-microscope, and the de-embedding the larva), starting at 1 dpi, we are able to report the time course of both absolute and relative regrowth for the 1–4 dpi for all experimental larvae of Fig. 5. Summed up, axon injury of 75 μm or closer to the soma reduces and below 20 μm even blocks regrowth of the M-axon. But regeneration of the M-axon is also clearly reduced at large distances of the injury from the soma. However, for axonal injury between 250 μm and 1250 μm from the soma regeneration was rapid and robust.

### Measuring functional regeneration in a single injured axon

**Fig. 6:**
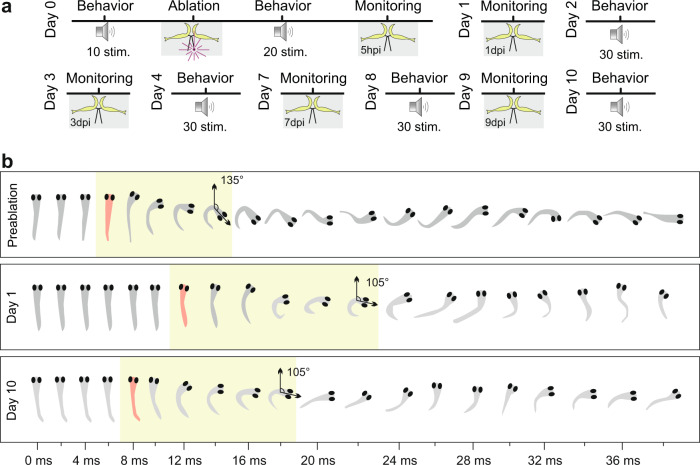
Using the M-cell driven C-starts to monitor functional recovery during axon regrowth. **a** Protocol used to monitor M-cell axon regeneration and C-start behavior over the course of 10 dpi in individual larvae. Gray areas indicate times during which larvae were fixed in low-melting agarose (10–20 min), otherwise larvae were allowed to swim freely. During the day at which specifically the GFP-filled M-axon was injured, a baseline of escape performance was first established and the immediate effect of the injury assessed. Then axonal growth was monitored on the indicated days and behavior always assayed on the subsequent day. During the behavioral assays each larva received 30 stimuli, with ten minutes between them. **b** Examples of escape responses recorded with digital high-speed video (3000 frames per second; every 6th frame shown; see SM2) in one individual larva prior, one day and ten days post-injury. Stimuli were given at time zero and latency (i.e., first notable movement of larva) is indicated by marking the larva red. The green boxes indicate the interval during which the larva bent maximally. Angles of turning are also indicated. These examples highlight the possibility that (i) a clear effect of injuring one single M-axon can indeed be detected and (ii) that aspects of the behavior (e.g., latency) might already be compensated in the days that follow after injury.

The recently discovered uniquely direct link between the functionality of the M-cell and the demonstrably live-saving short-latency escapes^32^ allows for a direct assay of how the regeneration of one single axon translates to the recovery of its function. Moreover, through careful examination of axon-morphology using two-photon microscopy, and interspersed behavioral testing, it is possible to monitor the recovery of function in parallel with axon regeneration at a temporal resolution of only one day. Figure 6a illustrates this approach in which axonal regrowth could be tested by embedding the larva and subsequent freeing the larva allowing to monitor its ability to elicit short-latency escapes. Based on the findings reported above we examined the speed of functional recovery after precisely targeted injury of the M-axon at 500 μm from its soma. At this distance axon regeneration would still be fast (see Fig. 5b) but also a sufficient number of motoneurons would be without command after the post-injury decay of the distal part of the M-axon. This should thus create a strong escape phenotype so that it would be easy to examine any subsequent recovery. Monitoring the time course of functional recovery would then allow us to assess the additional time needed for the regenerated axon to myelinate and to form new synapses (as suggested ref. ^19^) with motoneurons. Figure 6b provides examples from this approach. It shows images taken from digital high-speed video recordings of one individual larva in which axon regrowth and its escape starts had been followed for ten days. Slowed-down versions of the original videos are shown in SM2. These examples should prepare the quantitative analysis reported below of the M-axon specific injury on escape latency (i.e., start of C-bending after the stimulus at time zero) and its subsequent decline as the M-axon regrows. This illustrates (as will be substantiated below) that targeted injury of one specific M-axon causes a clearly detectable functional phenotype and that recovery of function can be monitored in parallel with the regeneration of one specific axon. The recordings also are shown to indicate that the injuries affect other aspects of the escapes, such as the time needed for bending into the typical C-shape (marked green in the examples of Fig. 6b), so that it will be possible to quantitatively monitor recovery of different aspects of function.

### Escape latency is fully restored in just 8 days

**Fig. 7:**
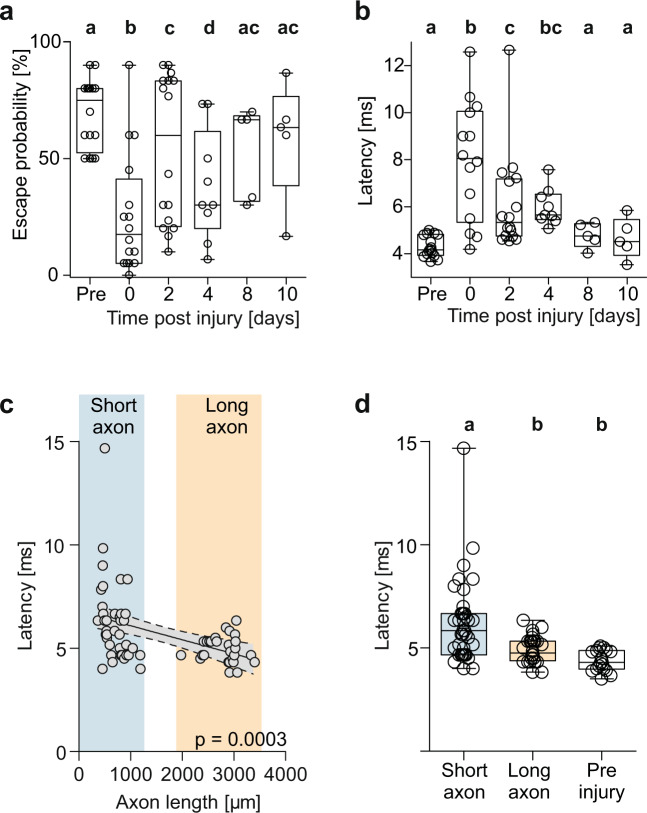
Rapid and complete functional recovery of escape probability and latency. **a** Boxplots to show the drop in escape probability after M-axon specific injury and subsequent recovery. ‘Pre’ denotes baseline before injury, and 0, 2, 4, 8, 10 dpi denote the days after injury when probability was determined. Each circle shows the escape probability of an individual larva. Significant differences are highlighted with different letters. Note that escape probability after 8 and 10 dpi has reached the preinjury level (glmm with multiple pairwise comparison (Tukey); *N* = 16). **b** Same analysis for the latency of the escape response with circles showing mean latency for each individual larva. Note that injury not only causes a highly significant increase in latency but also in variance (see text). After 8 and 10 dpi latency has been restored completely (lmm with multiple pairwise comparison (Tukey); *N* = 16). **c** Correlation between axon length, measured the day before, and escape latency. Slope differs significantly from zero *P* = 0.0003; R^2^ = 0.2), indicating continuous decrease in latency as the axon regrows (n = 68 latency medians taken at various lengths of the regenerating axon, *N* = 16 larvae). **d** Latencies from **c** but grouped according to stages in which the M-axon was still short (<1200 μm; *n* = 38), or already long (>1900 μm; *n* = 28) and compared to preinjury latency (*n* = 16). Circles show individual medians of latency for each group of axon length. Significant differences are highlighted with different letters (Kruskal–Wallis with Dunn’s multiple comparison post-hoc test. *P* < 0.001 for short axons vs. preinjury and *P* < 0.01 for long axons vs. short axons).

For the quantitative analysis of the recovery of function in parallel with the recovery of the M-axon, following targeted M-axon specific injury, we examined (i) the probability at which short-latency escapes were elicited by our standardized stimulus and (ii) the actual latency of the escapes. Escape probability was defined from the responses that occurred within an interval of 70 ms after the stimulus. Baseline response probability for our stimuli was 70 ± 4% (Fig. 7a; *N* = 16 larvae, total of *n* = 160 stimuli). At 1 dpi release probability had dropped dramatically (general linear mixed model with multiple pairwise comparisons (Tukey) vs. preinjury; *P* < 0.0001) to only 26 ± 6% (*N* = 16; *n* = 320). Response probability then increased again and after 8 dpi (53 ± 9%; *N* = 5, *n* = 150) was no longer significantly different from its preinjury values (*P* = 0.22) and significantly higher than the intermediate probability at 4 dpi (*P* = 0.0009; 38 ± 8%; *N* = 9, *n* = 270). We would also like to note, that prior to injury, escape response parameters of our larvae were similar as reported for intact larvae in other studies^24,38^. The larvae also responded as quickly to our acoustic stimulus as reported in earlier studies^32,43^, with a latency of 4.3 ± 0.1 ms (Fig. 7b; *N* = 16, *n* = 109). However, in the first 20 stimulations that followed at 1 dpi (see Fig. 6a) latency was significantly increased to 7.9 ± 0.7 ms (*N* = 16, *n* = 80; linear mixed model with multiple pairwise comparisons (Tukey); *P* < 0.0001). Latency, as probed by 30 stimulations per day for each larva, then declined over the course of the following 4 days. Already at 8 dpi latency had recovered completely and was no longer significantly different from its preinjury values (*P* = 0.91; *N* = 5 from *n* = 80) and significantly shorter than the intermediate latency values at four dpi (*P* = 0.008; *N* = 9 from *n* = 92). Interestingly, plotting median latency against axon length, measured in the same larvae on the day that immediately preceded each behavioral assessment (see Fig. 6a for the procedure), shows a relation between latency and axon length (Fig. 7c; linear regression R^2^ = 0.2; Difference of slope from zero: *P* < 0.0003; *n* = 66 axon lengths from *N* = 16). Latency tended to decrease as the axon regrew: Average latency measured when the axon was still short (i.e., its length below 1200 μm) was 6.2 ± 0.3 ms (Fig. 6d; *n* = 38 from *N* = 16) and differed from both latencies with long axons (length > 1900 μm; Kruskal–Wallis; *p* < 0.001) and latencies before injury (*p* < 0.0001). Remarkably, latency determined with long axons (>1900 μm) was 4.9 ± 0.1 ms (*n* = 28 from *N* = 10) and did not differ from latency before injury (4.4 ± 0.1 ms; *P* = 0.1; *n* = 16).

The injury not only increased median latency but also caused considerable variations in latency, as can be seen in the boxplots of Fig. 7d. The variance of response latency was significantly increased in the short M-axon state (i.e., following injury) both compared to the original state before injury (Brown–Forsythe; *P* = 0.0019) and also compared to when the axon had regenerated (‘long axons’; Brown–Forsythe; *P* = 0.0032). Interestingly, also the post-injury increase in variance in latency was restored completely in the long-axon regenerated state and did not differ significantly from the preinjury variance (Brown–Forsythe: *P* > 0.9).

Our data also allowed to examine an interesting additional aspect: By comparing the sigmoidal growth of the M-axon in the previous experiments without stimulation (i.e., Figs. 3, 4) and in the present experiments with induced escapes (Fig. 6) the ‘training’ effect of actually executing a response on axon regeneration can directly be tested. We were unable, however, to detect major changes between the sigmoidal axon growth curves obtained with and without stimulation (with stimulation: steepness s = 0.6; max. slope at 2.1 days; R^2^ = 0.97; *N* = 6; no difference from characteristic parameters of growth without stimulation: *P* = 0.21; Extra sum-of-square F test) suggesting that M-axon regrowth is independent of both the stimulation and of practical training (i.e., feedback from executing the behavior).

In summary, by measuring the regrowth of a single axon after precisely targeted injury of only this axon and by monitoring short-latency escapes that require the axon^32^ in the same larvae and over the course of several days we were able to demonstrate that escape probability, median escape latency and variation in escape latency recover extremely rapidly after an initial injury-induced impairment. These findings suggest that all processes that must occur in addition to axonal regrowth, such as myelination and appropriately targeted synaptogenesis occur rapidly and in sufficient quality to ensure rapid recovery of escape probability and latency.

### Evidence for additional slower processes

**Fig. 8:**
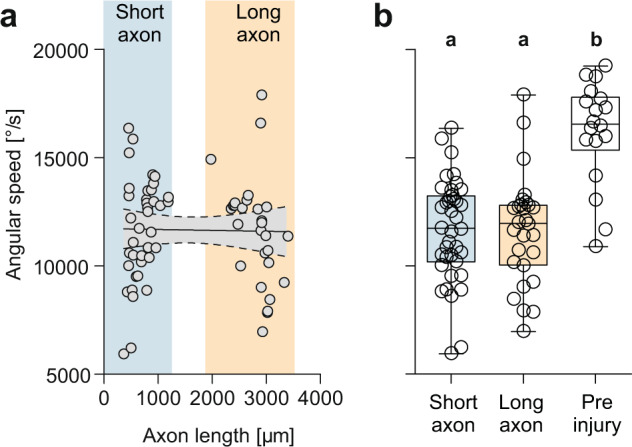
Evidence for additional slower processes that are not completed during axonal regrowth and recovery of latency. **a** Linear regression between median angular speed (i.e., bending angle divided by bending duration) and axon length 1 day before escapes were recorded. While escape latency had been completely restored to preinjury levels when axons had regenerated to the state marked as ‘long axon’, median angular speed of the escapes stayed constant, i.e., the slope of the regression line does not differ from zero (*P* = 0.88; *n* = 68 stages in *N* = 16 larvae). **b** Boxplots show angular speed before injury and when the axons had regrown to short (<1200 μm) or long axon length (>1900 μm). Circles show medians of angular speed for each axon length. Note absence of significant differences (highlighted with different letters; Kruskal–Wallis with Dunn’s multiple comparison post-hoc test) in angular speed of the different states during axon regrowth and difference at both stages from the angular speed level seen before injury (*P* < 0.0001). Data were obtained from the same escape responses (and same regeneration states) as in Fig. 7 that showed complete restoration of escape latency.

The speed at which probability and latency were restored during M-axon regeneration is impressive. It suggests that myelination of the regrown axons and targeted formation of synapses onto the motoneurons is sufficiently quick to rapidly initiate escapes after injury. However, it is unlikely that synapse formation and myelination should both be fully completed in just a few days. We therefore examined other aspects of the escape starts that might be indicative of additional slower processes. The effect of the injury on angular speed of the initial escape (taken simply as bending angle divided by bending duration, see Fig. 6b) turned out to be a good example. Plotting median angular speed against axon length (as measured in the same larvae on the day before, see Fig. 6a) shows that angular speed indeed remains diminished after injury and unchanged during the time needed for regrowth of the M-axon and for restoring latency (Fig. 8a; linear regression shows no significant difference from zero slope; *P* = 0.88; *n* = 66 axon lengths determined at different dpi in *N* = 16 larvae). Before injury angular speed was 16182 ± 560 °/s (*n* = 16). After specific M-axon injury it dropped to 11667 ± 378 °/s (Fig. 8b; *n* = 38 from *N* = 16) without significant recovery in the first 10 days after injury. When all regenerative states are considered in which the axon had grown beyond 1900 μm (i.e., the ‘long’ axons) average angular speed was still 11651 ± 471 °/s (*n* = 28 from *N* = 10) and thus significantly lower than prior injury (Kruskal–Wallis; *P* < 0.0001). The lack of recovery of angular speed therefore suggests that there are additional processes that require more fine-tuning as did the complete recovery of latency in just a few days.

## Discussion

Here, we show that it is possible to study functional recovery after targeted two-photon laser-injury of a single axon in larval zebrafish. This approach revealed that the M-axon can regenerate very rapidly and that essential functions of the behavior driven by this neuron^32^ are fully recovered in just days. As we report here, the regenerative capacity is not distributed uniformly across the axon and its decline after distant injury explains earlier reports of poor regeneration of the M-axon. The M-axon is therefore an unusually powerful system that allows simultaneous high temporal resolution monitoring of the recovery of motor function during the regeneration of an identified individual neuron in the vertebrate CNS after injury. Additionally, it shows one of the major factors seen currently as a potent leverage^33–40^ to understand mechanisms that enable or inhibit regeneration: at least in zebrafish larvae it exhibits an injury-site dependent switch that decides between rapid and functional regeneration or poor regeneration.

Our findings of rapid functional regeneration in the M-axon are particularly reassuring from a neuroethological point of view. Earlier work suggested that the M-axon regenerates slowly^14,24–27,33^ and that latency of escapes triggered by the M-axon never fully regenerate^30^. In larval zebrafish distal injury of the spinal cord, that also injured the M-axon, caused an increase in escape latency that did not recover naturally. It could, however, be made to recover when the growth of the M-axon was promoted to be similar as that of other spinal axons by application of high doses of cAMP^24^. Recent findings show that the Mauthner axon of both larva and adult zebrafish is required for short-latency escapes and the importance of these starts for survival^32^ makes it puzzling why especially the Mauthner axon should regenerate less efficiently than other axons in the spinal cord: Survival requires the M-axon to have a certain minimal length, so that a large number of motoneurons and a large fraction of trunk muscle can be rapidly activated^44,45^. The urgency for rapid regrowth of the M-axon and of rapid recovery of escape latency is thus particularly high after proximal injury. Evolution seems indeed to have equipped the M-axon not only with mechanisms for rapid regrowth but also with mechanisms that allow function to be also quickly restored. The speed of functional regeneration after injury in the most critical region of the axon is impressive, given that functional regeneration has otherwise been reported to occur over the course of weeks^31^, months^28,29,46^ or even years^30^. Our findings demonstrate two important aspects of functional recovery in the M-axon: Firstly, its regenerative power is not distributed homogeneously but is particularly high in the important middle section of the axon. Secondly, the evolutionary pressures on mechanisms needed to regain function are not homogeneously distributed across all functions (see Fig. 8). These two complexities, together with the unusual high-resolution methods available, probably make the M-axon an even more important system, at least in larval zebrafish, as it was previously: It involves both rapid and slow processes, which makes it interesting to explore what could be the switch that decides between the two. And its distant-dependent regeneration makes it a good model in which to study how distance-dependent processes interfere with regeneration, an approach that is currently seen as very promising^33–40^.

Mapping of the regenerative capacity across the axon revealed that the M-axon offers the possibility to explore what aspects of distance are critical in either inhibiting (at large distance from the soma), promoting (at short distance from the soma) or preventing (at very close distance from the soma) axonal regrowth in one single neuron of the vertebrate CNS. Although the focused two-photon laser beam demonstrably did not affect the soma directly at the closest (20 μm) distance we have explored, the soma died off in 25% of the cases (see Fig. S1). In all remaining cases the axon also did not regrow. In contrast, at larger distance, the regenerative capacity is high and it decays only at larger distance from the soma. There thus appear to be at least two processes that account for the optimum-curve like distribution of regenerative capacity (see Fig. 5): First, the reduced capacity after injury very close to the soma might be due to an initial axonal retraction response (also known as axonal dieback) that has been suggested (after injury set with different methods) in other systems^47,48^. Second, additional processes must account for the decay of regenerative capacity farther away from the soma. As argued above this explains the poor regeneration in earlier work^14,24–27,33^. Based on the earlier finding of the effect of additional doses of cAMP on regeneration of the M-axon we suggest that distance-dependence might largely be due to processes that affect the expression of regeneration associated genes (RAGs) whose regulation depends on cAMP as a second messenger^49,50^. In such a scenario the expression of RAGs would be reduced after distal injury, resulting in poor regeneration that can partly be increased by adding cAMP. If this was correct, then the signaling pathway that relates local injury-site signals to RAG expression would be a promising target. We therefore hope that using two-photon microscopy for targeted injury of a single M-axon in zebrafish larvae and the unusually direct relation to short-latency escapes will prove to be valuable tools for understanding the nature of the distance-dependency and for exploring the many processes that must be orchestrated to achieve functional recovery. Monitoring the expression of particularly interesting genes after proximal and distal injury would be possible in this system^51,52^ and could be instrumental for identifying key processes that may be shared between mammalian and nonmammalian vertebrates^7,16,52–54^.

## Methods

### Animal Care

Adult zebrafish (*Danio rerio*) were kept in a fish housing system at 28.5 °C. Embryos were raised at 28.5 °C on a 12 h:12 h light/dark cycle in E3-Medium (5 mM NaCl, 0.17 mM KCl, 0.33 mM CaCl_2_, 0.33 mM MgSO_4_x7H_2_O, 10^−5^% Methylene Blue in dH_2_O). Experiments were according to the German law on animal welfare and approved by state authorities. At the beginning of each experiment larvae were 5 dpf (days post fertilization). We used a new *Ca-Tol-056* strain that was generated by crossing the *Tol-056*-strain^55^ (Et(T2KHG)zf206) and the pigmentless *casper* strain^56^ (*mitfa*^*w2/w2*^*;mpv17*^*a9/a9*^), which allows better penetration of the laser in the tissue. The *Tol-056*-line is an enhancer trap line that expresses GFP in the Mauthner cell and in a limited number of other, sufficiently distant, CNS neurons. The GFP labeling allows targeted M-axon specific injury using a two-photon laser and the monitoring of the state of the regenerating axon.

### Systemic SCI including M-axon injury

Five dpf larvae were anesthetized in 0.04% ethyl 3-aminobenzoate methanesulfonate (MS-222) in dH_2_0 for ten minutes. Larvae were then transferred to a small petri dish and embedded, while lying on the side, in 1.5% low-melting-point agarose (LMP agarose). For systemic injury we used a pulled glass microelectrode (GB150F-8P, Science Products GmbH, Hofheim) that was broken and moved to the desired location along the most dorsal part of the notochord. Fish were injured at distances from the M-cell soma between 523 ± 31 μm (slightly caudal to the swim bladder), between 1365 ± 38 μm or between 1599 ± 31 μm (around the level of the 15th somite) and 1760 ± 38 μm. Afterwards larvae were removed from the agarose and transferred in E3-medium at 28.5 °C to recover from the procedure and were fed with dried food or paramecia.

### Laser-induced (targeted) injury of Mauthner axons

Five dpf Zebrafish larvae were anesthetized in 0.04% 3-aminobenzoic acid ethyl ester (MS-222) in dH_2_O for 10 min and embedded in 1.5% LMP agarose (Sigma–Aldrich, St. Louis) with their dorsal side up in a small petri dish that was placed under the multiphoton microscope. We used an 80 MHz titanium:sapphire multiphoton excitation laser (Mai Tai DeepSee, Spectra Physics, Stahnsdorf) tuned to 900 nm that was introduced into a laser-scanning microscope (Leica TCS SP5 II with HCX IRAPO L, 25.0 × 0.95 Water, Leica Microsystems CMS GmbH, Mannheim). To check if the M-cell was appropriately developed and intact we first made a z-stack (50–100 μm with 0.5–2 μm for each focal plane) of both M-cell axons (592 × 592 μm) prior to injury. For targeted Mauthner axon (M-axon) injury we identified the M-cell via its large soma, using low laser energy (to avoid tissue damage) and maximally zoomed into the axon area (9.23 × 9.23 μm) a set distance (see below) away from M-soma. To avoid tissue damage in the direct vicinity of the M-axon we set a region of interest (ROI), that only covered the axon (size adjusted from 4 to 6 × 9.23 μm, depending on axon thickness). Laser energy was then set to the maximum level. Injury usually was successful after one single scan over the ROI or when the homogenous fluorescence shifted to a more heterogeneous fluorescence after the bursting of the M-axon, with high fluorescent spots all over the image (between one and five seconds or up to five scan repeats; each image 592 × 592 μm; scan speed 400 Hz). After this procedure, larvae were removed from the agarose, were fed with dried food (Novo Tom Artemia, JBL, Neuhofen) or paramecia and were allowed to swim freely in E3-medium at 28.5 °C until the next laser scan or a behavioral session was scheduled. We injured the M-axon at one out of eight possible distances (19 ± 0.3 μm, 75 ± 1 μm, 275 ± 1 μm, 495 ± 17 μm, 743 ± 2 μm, 1283 ± 2 μm, 1775 ± 4 μm, and 2296 ± 7 μm).

### Measuring regrowth of the Mauthner axons

To monitor and measure the axon length post-injury we anesthetized and embedded the fish again as described above at different days post-injury (dpi; indicated in the figures and figure legends). Larvae were anesthetized as briefly as necessary (30–60 s) to just immobilize them during embedding. Thirty-five percent of the larvae already showed weak movement at the end of embedding but no attempts were needed to straighten these larvae or to increase anesthesia because our measurements did not require the axons to be straight. Embedded larvae were placed under the microscope to create stacks (50–100 μm vertical depth with 0.5–2 μm for each focal plane) of M-cell bodies and remaining M-axons. Because areas scanned were 592 × 592 μm, between one and six images were needed for a complete image of the whole M-cell, depending on the length of the remaining axon. To take these images, starting rostrally (i.e., with the soma) we used a remote control (Keypad SM7; Luigs & Neumann) to move the microscope table in fixed steps of 535 μm. The individual images were then stitched together using the Fiji software. First, we created a maximum intensity Z-projection of each stack (standard deviation type) and saved every image as a TIF file. We then used the Fiji plugin ‘Pairwise stitching’^57^ (Fusion method: Linear blending and y-value: 535 μm) to stitch the individual images until an image of the complete axon was achieved. We then applied the ‘segmented line’ tool to measure axon length from the axon hillock to the most caudal axon stump (note that axons initially formed multiple sprouts). We found that survival of treated larvae was improved by keeping them in groups of four or five larvae in small petri dishes (Ø 60 mm) and that identifying the individual larvae was easy, based on their individually distinct fluorescence pattern of cells marked additionally to the Mauthner cells (that are always labeled). However, we note that larvae that were to be used to combined monitoring of axon recovery and functional assays were always kept individually throughout the whole experiment.

### Acoustical stimulation of zebrafish larvae

Six zebrafish larvae (five treated and one control) were transferred into six petri dishes (Ø 35 mm) with 2 ml E3-medium (5 mM NaCl, 0.17 mM KCl, 0.33 mM CaCl_2_, 0.33 mM MgSO_4_x7H_2_O, 10^−5^% Methylene Blue in dH_2_O). The petri dishes were arranged in a hexagonal pattern on a transparent acryl glass plate around a vibrational speaker (PocketBoom, Mobile Fun Limited, Hamburg) that was attached to the top of the glass plate, so that every larva received approximately the same stimulus strength. A function generator (TGP110 10 MHz, AIM-TTi, Huntingdon) delivered a single rectangular pulse (100 μs) with an amplitude of 800 mV to the vibrational speaker. Escape responses were recorded by monitoring the dishes from below, using a high-speed camera (3000 fps; FASTCAM APX RS, Photron, Pfullingen) that was set so as to automatically start recording 10 ms prior to stimulus and to stop 130 ms after the stimulus had ended. The arrangement was homogeneously illuminated by arranging a 500 W halogen spotlight above the setup. The temperature was controlled to always remain between 27 and 28 °C. On the first day we stimulated larvae 10 times prior injury and 20 times post-injury. Controls were treated to mimic this procedure, i.e., they also were stimulated 30 times a day, with 10 initial stimulations, an embedding that lasted similarly as in the experimental animals, and then the final 20 stimuli. Escape parameters of controls were constant during the complete time course of 10 days (e.g., latency of controls was 4.6 ± 0.15 ms; 214 escapes from two larvae). After the first day, larvae always received 30 stimuli with an interstimulus interval of 10 min. At the end of each day larvae were transferred in fresh E3-medium with dried food or paramecia at 28.5 °C. High-speed videos were analyzed using Fiji.

### Statistics and reproducibility

Statistical analyses were run on GraphPad Prism^®^ 5.01 (GraphPad Software, Inc.). A sigmoidal nonlinear fit (variable slope) was used to model the time course of axon regrowth because R^2^ was always higher than 0.70 (except in the condition without regrowth, i.e., at systemic SCI 1600–1700 μm from the M-cell somata). Differences in the fits obtained for regrowth after systemic SCI and after laser-induced injury were compared using the extra sum-of-square F test. The regular growth of the axon in intact larvae at the age of our experimental larvae is also explicitly displayed (to demonstrate completeness of regeneration) and best described by linear fits. A mixed model approach was performed using R Studio version 1.2.1335/R version 3.6.0 (R Core Team, 2019) and the lme4 package. We fitted two separate models (1) with escape probability and (2) with latency as the response variable. For each model we tested whether (1) the escape probability (glmm, binomial model) and (2) the escape latency (lmm) was affected by the days post-injury (dpi). Thus, dpi was a fixed factor and as this was a repeated-measures experiment we included fish identity as a random factor to account for variation within individual fish. Post-hoc analysis for both models was assessed by using the ‘emmeans’ package in R, with *p*-values adjusted for multiple comparisons (Tukey method).

For correlation between latency/angular speed and axon length we ran a linear regression analysis with a 95% confidence band of the best-fit line. We only created medians and plotted latency and angular speed if at least two responses were elicited at this axon length.

To compare axon length 4 dpi after injuries (SCI) at either 1365 ± 38 μm or 1760 ± 38 μm distance from the soma we used the Mann–Whitney U test. To compare relative regrowth of axons at 4 dpi as well as latency and angular speed of the escapes, normality was checked and Kruskal–Wallis-tests were run (with Dunn’s multiple comparison for post-hoc test). Boxplots show the interquartile range of 25–75% with whiskers from minimum to maximum. Circles in boxplots show individual medians.

### Reporting summary

Further information on research design is available in the Nature Research Reporting Summary linked to this article.

## Supplementary information

Supplementary Movie 1

Supplementary Movie 2

Supplementary Information

Supplemantary Data 1

Supplemantary Data 2

Supplemantary Data 3

Supplementary Data 4

Supplemantary Data 5

Supplemantary Data 6

Supplemantary Data 7

Description of Additional Supplementary Files

Reporting Summary

## Data Availability

The data that support the findings of this study are available from the corresponding authors upon reasonable request.
