# Supplementary material for: High-resolution mapping of injury-site dependent functional recovery in a single axon in zebrafish
Source: Commun Biol. 2020 Jun 12;3:307. doi: 10.1038/s42003-020-1034-x (PMC7293241; doi:10.1038/s42003-020-1034-x)
Supplement: Supplementary file 3 — Supplementary Information [file 42003_2020_1034_MOESM3_ESM.pdf]

Supplementary Information for

High-resolution mapping of injury-site dependent functional recovery in a single axon in zebrafish

corresponding authors: Stefan Schuster, Alexander Hecker
Email: stefan.schuster@uni-bayreuth.de, alexander.hecker@uni-bayreuth.de

This PDF file includes:

Supplementary Figure 1
Supplementary Figure 2

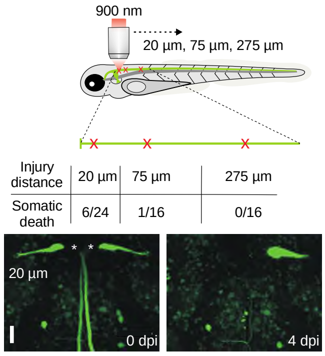

Supplementary Figure 1. The effect of M-axon injury very close to the soma. Illustration of experiments with precisely targeted Mauthner axon injury close to the soma, starting at 20 µm distance (19 ± 0.3 µm, top panel). The table reports the number of instances in which the soma died within one day vs. the total number of injured axons. When the soma survived, then regeneration did not start earlier than after 4 dpi. The images show an example of an experiment, with two-photon-laser z-stacks to monitor cell state directly after injury (0 dpi; injury sites indicated by two asterisks) and at 4 dpi. Scale bar = 25 µm.

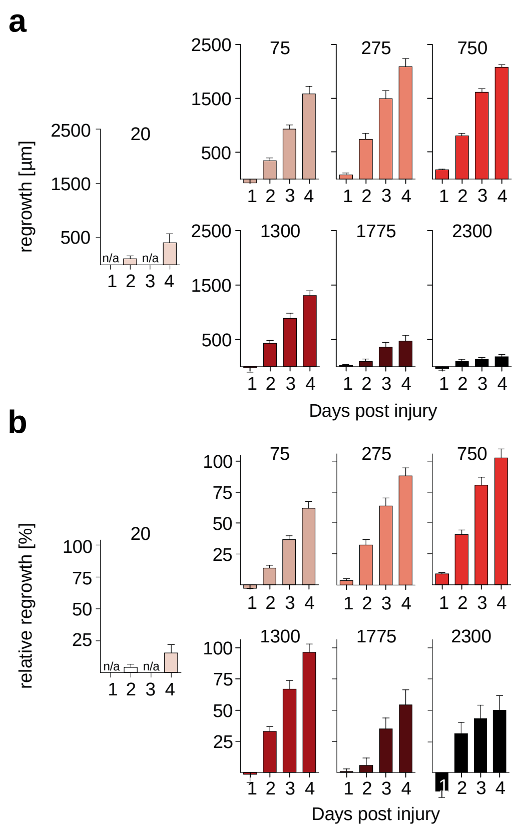

Supplementary Figure 2. The positional dependency or both absolute and relative regrowth is already evident earlier than 4 days after injury.

a, b Absolute (a) and relative (b) regrowth of the M-axon for several distances of targeted M-axon specific laser injury at set distances from the soma. Regrowth is reported for the first four days after injury (N = 10 – 14 M-cells). Relative regrowth is absolute regrowth normalized to the virtual regrowth (relative to a fictive injury at the same position) in equally aged control siblings (N = 8 M-cells) (see Fig. 5). Note the negative regrowth (retraction of the axon stump) on the first dpi that can be seen across the whole spectrum of injury distances.
