# Supplementary material for: High-resolution mapping of injury-site dependent functional recovery in a single axon in zebrafish
Source: Commun Biol. 2020 Jun 12;3:307. doi: 10.1038/s42003-020-1034-x (PMC7293241; doi:10.1038/s42003-020-1034-x)
Supplement: Supplementary file 11 — Description of Additional Supplementary Files [file 42003_2020_1034_MOESM11_ESM.pdf]

Description of Additional Supplementary Files

Supplementary Movie 1: Time-lapse movie showing the regeneration of Mauthner cell axons after proximal injury.
After embedding a zebrafish larva of the transparent line *Ca-Tol-056*, with GFP labelled Mauthner neurons, the M-axons are specifically cut with a two-photon laser, 500 µm from the soma. Subsequently z-stacks (150 images) were taken – without interruption – every ten minutes for a total of 66 hours post injury. The movie starts with a countdown (top left) during which an initial impression of the intact M-axons can be gained and in which asterisks indicate the future injury sites. At the end of the countdown, the laser causes the degeneration of the M-axon around the injury site. A time scale (top left) indicates hours post injury (hpi). A scale bar is shown at the bottom left. Wallerian degeneration of caudal axon segments starts at around 21 hpi. The regeneration of the axons starts between eight and ten hpi. Both axons initially regenerate by forming multiple sprouts. However, the axon seen on the right side quickly reduces the number of sprouts and only the main axon remains at 65 hpi.

Supplementary Movie 2: High-speed videos showing rapid functional recovery after proximal M-axon injury.
The uniquely direct link between functionality of the M-axon and the short-latency escape C-starts[32] allows an assay of functional recovery at high temporal resolution while the regrowth of the axon can also be monitored in the same individual larvae using two-photon microscopy (see SM1). The video gives examples of digital high-speed videos of escape C-starts of one individual larva taken prior (left), one day (middle) and 10 days post injury (right). Videos were taken at 3000 frames per second and are slowed down to 5 fps. The escapes are those shown schematically in Fig. 6. A time stamp is shown in the lower left and stimuli were given at time zero. After injury, escape latency increased from 5 ms to 11 ms (1 dpi) but dropped to 7 ms at 10 dpi.

Supplementary Data 1-7

All source data underlying the graphs and charts presented in all main figures are available as Supplementary Excel files (Fig 1e.xlsx; Fig 1f.xlsx; Fig 2. xlsx; Fig 3c.xlsx; Fig 4.xlsx; Fig. 5b.xlsx; Fig 5c.xlsx; Fig. 7.xlsx; Fig. 8.xlsx).
