# Supplementary material for: High-resolution mapping of injury-site dependent functional recovery in a single axon in zebrafish
Source: Commun Biol. 2020 Jun 12;3:307. doi: 10.1038/s42003-020-1034-x (PMC7293241; doi:10.1038/s42003-020-1034-x)
Supplement: Supplementary file 12 — Reporting Summary [file 42003_2020_1034_MOESM12_ESM.pdf]

nature research

Corresponding author(s): Stefan Schuster, University of Bayreuth

Last updated by author(s): May 20, 2020

Reporting Summary

Nature Research wishes to improve the reproducibility of the work that we publish. This form provides structure for consistency and transparency in reporting. For further information on Nature Research policies, see Authors & Referees and the Editorial Policy Checklist.

Statistics

For all statistical analyses, confirm that the following items are present in the figure legend, table legend, main text, or Methods section.

n/a	Confirmed	
☐	☒	The exact sample size (n) for each experimental group/condition, given as a discrete number and unit of measurement
☐	☒	A statement on whether measurements were taken from distinct samples or whether the same sample was measured repeatedly
☐	☒	The statistical test(s) used AND whether they are one- or two-sided<br>*Only common tests should be described solely by name; describe more complex techniques in the Methods section.*
☒	☐	A description of all covariates tested
☐	☒	A description of any assumptions or corrections, such as tests of normality and adjustment for multiple comparisons
☐	☒	A full description of the statistical parameters including central tendency (e.g. means) or other basic estimates (e.g. regression coefficient) AND variation (e.g. standard deviation) or associated estimates of uncertainty (e.g. confidence intervals)
☐	☒	For null hypothesis testing, the test statistic (e.g. F, t, r) with confidence intervals, effect sizes, degrees of freedom and P value noted<br>*Give P values as exact values whenever suitable.*
☒	☐	For Bayesian analysis, information on the choice of priors and Markov chain Monte Carlo settings
☒	☐	For hierarchical and complex designs, identification of the appropriate level for tests and full reporting of outcomes
☒	☐	Estimates of effect sizes (e.g. Cohen's d, Pearson's r), indicating how they were calculated

Our web collection on statistics for biologists contains articles on many of the points above.

Software and code

Policy information about availability of computer code

Data collection	Videos were acquired using a high-speed camera (FASTCAM APX RS; Photron). M-cell images were acquired using an 80 MHz titanium:sapphire multiphoton excitation laser (Leica TCS SP5 II with HCX IRAPO L, 25.0x0.95 Water, Leica Microsystems)
Data analysis	Fiji (ImageJ 1.48s) was used for Data anaylsis; GraphPad Prism (v5.01; 2007) and R (1.2.1335/R version 3.6.0) were used for statistical analysis.

For manuscripts utilizing custom algorithms or software that are central to the research but not yet described in published literature, software must be made available to editors/reviewers. We strongly encourage code deposition in a community repository (e.g. GitHub). See the Nature Research guidelines for submitting code & software for further information.

Data

Policy information about availability of data

All manuscripts must include a data availability statement. This statement should provide the following information, where applicable:
- Accession codes, unique identifiers, or web links for publicly available datasets
- A list of figures that have associated raw data
- A description of any restrictions on data availability

All data will be available in available datasets or upon reasonable request to the Schuster laboratory after publication.

Field-specific reporting

Please select the one below that is the best fit for your research. If you are not sure, read the appropriate sections before making your selection.

☒ Life sciences ☐ Behavioural & social sciences ☐ Ecological, evolutionary & environmental sciences

Life sciences study design

All studies must disclose on these points even when the disclosure is negative.

Sample size	No sample size pre-determination was performed. Samples size was chosen based on previous experience that would be sufficient to achieve statistical significance.
Data exclusions	No data were excluded.
Replication	All attempts of replication were succesful. Escape response anaylsis and Mauthner axon injuries showed similar results with other zebrafish larvae during similar experiments.
Randomization	Larvae were always randomly positioned in the experimental set-up during behavioural observations.
Blinding	The experimenter first analysed behavioural parameters and only afterwards measured the axon lengths of the zebrafish larvae.

Reporting for specific materials, systems and methods

We require information from authors about some types of materials, experimental systems and methods used in many studies. Here, indicate whether each material, system or method listed is relevant to your study. If you are not sure if a list item applies to your research, read the appropriate section before selecting a response.

Materials & experimental systems

n/a	Involved in the study	
☒	☐	Antibodies
☒	☐	Eukaryotic cell lines
☒	☐	Palaeontology
☐	☒	Animals and other organisms
☒	☐	Human research participants
☒	☐	Clinical data

Methods

n/a	Involved in the study	
☒	☐	ChIP-seq
☒	☐	Flow cytometry
☒	☐	MRI-based neuroimaging

Animals and other organisms

Policy information about studies involving animals; ARRIVE guidelines recommended for reporting animal research

Laboratory animals	We used zebrafish larvae (Danio rerio). We generated the zebrafish strain (called Ca-Tol-056) by crossing the strain Tol-056 with the pigmentless casper-strain (mitfaw2/w2;mpv17a9/a9). It is not possible to determine the sex in zebrafish larvae.
Wild animals	This study did not involve wild animals
Field-collected samples	This study did not involve field collected samples.
Ethics oversight	All experiments were approved according to the German law on animal welfare. (55.2 DMS-2532-2-414)

Note that full information on the approval of the study protocol must also be provided in the manuscript.
